# Prevention regulatory focus, desired cultural tightness, civic moral disengagement, and emotional reactions to normative daily transgressions: a serial mediation model among adults in Italy

**DOI:** 10.3389/fpsyg.2024.1340152

**Published:** 2024-05-09

**Authors:** Conrad Baldner, Antonio Pierro

**Affiliations:** Department of Social and Developmental Psychology, Sapienza University of Rome, Rome, Italy

**Keywords:** cultural tightness, prevention focus, moral disengagement, norm violations, transgression

## Abstract

Why do people have positive or indifferent reactions to norm violations? The present research hypothesized that individuals who focus on the avoidance of negative outcomes, for example punishments for rule violations, (i.e., a prevention focus) are hypothesized to also have a desire for rigid and clear norms (i.e., desired cultural tightness) as well as punishments for norm violations. Such norms and punishments narrow accepted behavior and, if clearly communicated, can limit rule violations. Consequently, individuals who desire higher levels of cultural tightness should be less likely to justify poor citizenship behavior (i.e., civic moral disengagement) as this behavior is antithetical to desired cultural tightness. Finally, such individuals should also be more likely to react negatively to norm violations. Data for the present study was conducted in Italy. A total of 1,181 participants were included in the analysis; participation requirements were that they be adults who were residents of Italy. Participants completed self-report measures of the prevention focus, desired cultural tightness, civic moral disengagement, and reactions to general norm violations (e.g., exceeding the speed limit, vandalism). The hypothesized serial mediation model was supported. This model can help explain why individuals can fail to react to “everyday” norm violations, as well societal-level violations (e.g., failing to respect hygienic standards during the COVID pandemic). It also calls on the need to develop mass communication approaches that can influence individuals’ prevention focus on a large scale, as this can have downstream effects of reactions to such violations.

## Introduction

Why do some people seemingly not care when others break social norms? Specifically, we aim to examine if a prevention regulatory focus, or a sensitivity to negative outcomes (Higgins et al., 1997), has an effect on reactions to social transgressions through the effects of desire for cultural tightness, or the desire for rigid social norms and consequent punishments (Mula et al., 2021), and civic moral disengagement, or the tendency to justify non-involvement in good citizenship (Caprara et al., 2009). If supported, this model could advance our knowledge on the general motivations for disengagement which could be applied to various specific contexts. Data was collected in Italy which, according to Gelfand et al. (2011), scores near the average on societal-level tightness, relative to a sample of 33 countries.

According to Regulatory Focus Theory (Higgins et al., 1997), individuals can be described in terms of two foci: a promotion focus, or the focus on the presence or absence of rewards or positive outcomes, and a prevention focus, or the focus on the presence or absence of punishments or negative outcomes. Individuals who are primarily characterized by a prevention focus are more likely to be concerned with the avoidance of negative outcomes. Consequently, they are more likely to seek to avoid punishments than they are to seek to gain rewards. For instance, recent research has shown that a prevention focus is associated with greater acceptance of COVID regulations (Hartmann and Müller, 2023). This should not as a surprise considering that COVID only presented individuals with potential punishments but not potential rewards. However, the prevention focus has also been found to be related to other constructs which may perhaps be less obvious. For instance, Mula and Pierro (2022) found that the prevention focus was also related to preference for a “tight” culture, or one in which that has rigid laws and social norms, and in which violations of these norms are met with stiff consequences. Likewise, individuals with a prevention focus and who perceived more tightness in their culture also have higher life satisfaction (Contu et al., 2023). The rationale that underlies this relationship is that these “tight” societies can be perceived to be able to prevent rule violations via stricter punishments. This should be attractive to individuals with a prevention focus as they tend to avoid such punishments.

The theory of cultural tightness (or looseness) was developed by Gelfand et al. (2006) in order to explain differences in norms and punishments at the cultural level. For instance, tight nations tend to have high population density and territorial threats (Gelfand et al., 2011); tight states in the US tend to have more ecological and human-made threats (Harrington and Gelfand, 2014). At the national level, cultural tightness was found to be related to fewer deaths due to COVID, perhaps because tight nations are more likely to cooperate under threat (Gelfand et al., 2021). Although tightness at the national or regional level is an interesting predictor, it is clearly not a good fit for our scope: individual-level prevention focus is very unlikely to be exogenous to national-level tightness. However, an interesting feature of tightness is that individuals can desire a particular amount of tightness or looseness independent of whatever level of tightness or looseness that exists in their nation, region, etc. Individual-level desire for tightness or looseness, independent of the cultural level, has been found to predict negative reactions to organizational norm violations (Mula and Pierro, 2022), negative reactions to COVID norm violations (Baldner et al., 2022), and lower levels of moral disengagement (Di Santo et al., 2024). The rationale underlying these findings is that a desire for rigid norms is contrary to both committing norm violations as well as justifications for such violations (e.g., moral disengagement). In the context of our research, it is reasonable to hypothesize that the desire for tightness would predict lower levels of moral disengagement.

Moral disengagement was initially identified by Bandura et al. (1996); in general, it refers to justifications for actions which otherwise would be considered to be morally wrong. For example, “boys will be boys” and “nothing I can do will make a difference” have been used to justify—to morally disengage from—different types of wrongdoing. Different modes of moral disengagement have been identified by researchers (e.g., diffusion of responsibility) and it has been studied in various contexts. Caprara et al. (2009) identified civic moral disengagement as a particular context in which individuals justify disengagement from good citizenship (e.g., refraining from littering, vandalism, traffic violations). That civic moral disengagement would be associated with less negative reactions to norm violations is consistent with the idea of moral disengagement. Similar results for the work context have been uncovered by Fehr et al. (2019). Consequently, we expect that civic moral disengagement will be related to less negative reactions to norm violations.

## The present research

The present research aims to understand how moral disengagement can be decreased and aversion to normative transgressions can be increased. The research proposes a serial mediation model that links prevention regulatory focus, desired cultural tightness, civic moral disengagement, and reactions to normative daily transgressions.

**FIGURE 1:**
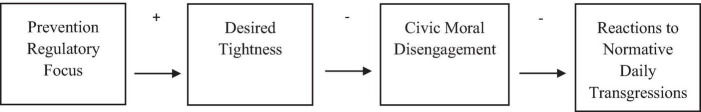
The theoretical model: the hypothesized effect of prevention regulatory focus on reactions to normative daily transgressions sequentially via desired tightness and reactions to normative daily transgressions.

The hypothesized relationships (see Figure 1) are as follows: (1) prevention focus should raise a desire for greater tightness, consistent with previous results (Jackson et al., 2019; Mula et al., 2021; Mula and Pierro, 2022); (2) people who desire tightness are more capable of self-regulation and more careful to behave in a socially acceptable manner (Mula et al., 2021; Baldner et al., 2022) and thus should be less likely to adopt moral disengagement strategies, whereas (3) moral disengagement strategies should be negatively associated with hostile reactions to normative transgressions.

## Method

### Participants and procedures

Data were collected from researchers and collaborators using snowball sampling. The eligibility criteria to participate in this research were to be at least 18 years old and to reside in Italy. A Monte Carlo power analysis suggested to collect a sample size ≥421 to achieve a minimum sufficient power of 0.80 assuming small to medium effects in a serial mediational model (Schoemann et al., 2017). A total of 1,181 participants (68.8% women), recruited through social networks, volunteered to participate in the study and provided explicit informed consent. Their mean age was 33.76, (*SD* = 14.32). A total of 5.9% of the participants had a middle school education or lower, 46% had a high school education, 28.2% had a bachelor’s degree, 19% had a master’s degree, and 0.9% had a PhD. In addition, 6.7% of the participants resided in northern Italy, 59% in central Italy, and 34.3% in southern Italy and the Island regions. Participants completed an online survey comprising the following measures and presented in this order as follows: social desirability, prevention regulatory focus, desired tightness, civic moral disengagement, and reactions to normative daily transgression. All study materials were presented in Italian and completed by participants in an online questionnaire. Written informed consent was obtained from all participants and their anonymity was guaranteed.

### Measures

#### Prevention regulatory focus

We used the Italian version of the Prevention Focus scale from the Regulatory Focus Questionnaire (RFQ, Higgins et al., 2001), composed of five items (e.g., “Not being careful enough has gotten me into trouble at times,” reverse scored). Ratings are made on a five-point Likert scale ranging from 1 (never or seldom) to 5 (very often). A composite score was computed by averaging across responses to the items (Cronbach’s α = 0.71). Values for kurtosis and skewness were −0.10 and −0.50, respectively. These values are sufficient, given the size of the sample, according to Kim (2013).

#### Desire for cultural tightness

Desire for cultural tightness was measured by asking participants if their place of residence should have “*loose*” vs. “*tight*” characteristics (Mula et al., 2021; Baldner et al., 2022). Specifically, they responded to five questions on a scale anchored from “1” to “9,” where high scores indicated high desire for cultural tightness. Example items are: “My place of residence should…”“1” = “*Have flexible social norms*” vs. “9” = “*Have rigid social norms*”; “1” = “*Be tolerant of people who violate the rules*” vs. “9” = “*Be intransigent with people who violate the rules.*” The reliability was good (Cronbach’s α = 0.88). Values for kurtosis and skewness were −0.38 and 0.015, respectively. These values are sufficient, given the size of the sample, according to Kim (2013).

#### Civic moral disengagement

Civic Moral Disengagement was measured via a brief version of the Civic Moral Disengagement Scale developed by Caprara et al. (2009) containing eight items, one for each of the eight moral disengagement mechanisms proposed by Bandura et al. (1996) and Bandura (2002). Examples of item are “It is not the fault of drivers if they exceed the speed limit since cars are made to go at high speeds,” “There is no sense in blaming individuals who evade a rule when everybody else does the same thing.” Items were rated on a five-point Likert scale, from “1” (*Completely disagree*) to “5” (*Completely agree*). A composite score was computed by averaging across responses to the items (Cronbach’s α = 0.77). Values for kurtosis and skewness were 1.47 and 2.72, respectively. These values are most likely elevated, according to Kim (2013), and indicate that most participants had low levels of moral civic disengagement.

#### Reactions to normative daily transgressions

Reactions were assessed with 3 adapted items derived from the scale proposed by Pepitome (1981) aimed at measuring the emotional reaction of participants to everyday normative violations (exceeding the speed limits while driving; disturbing the peace; carrying out acts of vandalism). Participants were asked to indicate their most likely emotional reaction (i.e., 1 = “*approval*,” 2 = “*indifference*,” 3 = “*contrary*,” 4 = “*anger*,” 5 = “*violent rage*”) in response to the above behaviors that could be carried out by others. A composite score was computed by averaging across responses to the items (Cronbach’s α = 0.69). Values for kurtosis and skewness were −0.36 and 0.56, respectively. These values are sufficient, given the size of the sample, according to Kim (2013).

#### Social desirability

To control for socially desirable responding, we used the following two items: “I have never been late for an appointment or work,” “I have never hurt another person’s feelings.” Responses were rated on a 6-point Likert scale from 1 (“*Strongly disagree*”) to 6 (“*Strongly agree*”). These items were developed by Pierro and Kruglanski (2005). The items positively and significantly correlated with each other (*r* = 0.30, *p* < 0.001) and were averaged to form a social desirability score.

#### Demographics

Participants were also asked as to their gender, age, and education level (i.e., Middle School, High School, Bachelor’s Degree, Master’s Degree, Ph.D.). These variables were entered as covariates.

## Results

### Preliminary results

Descriptive statistics and bivariate correlations between variables are reported in Table 1. As observed, prevention regulatory focus significantly and positively correlated with both desired tightness (*r* = 0.162, *p* < 0.001) and emotional reactions to normative transgression (*r* = 0.079, *p* < 0.01) and significantly and negatively correlated with civic moral disengagement (*r* = −0.240, *p* < 0.001); desired tightness correlated significantly and negatively with civic moral disengagement (*r* = −0.119, *p* = 0.001) and significantly and positively with emotional reactions to normative transgression (*r* = 0.219, *p* < 0.001). Finally, civic moral disengagement negatively correlated with emotional reactions to normative transgression (*r* = −0.233, *p* < 0.001).

**TABLE 1:** Descriptives and bivariate correlations (*N* = 1181).

	Age	Gen	Edu	Prev	DT	CMD	RD	M (SD)
Age	–							33.76 (14.32)
Gen	-0.033	–						–
Edu	-0.038	0.067*	–					–
Prev	0.132***	0.133***	0.064*	(0.71)				3.19 (0.79)
DT	0.115***	0.105***	-0.036	0.162***	(0.88)			6.25 (1.58)
CMD	-0.111***	-0.184***	-0.088	-0.240***	-0.119***	(0.77)		1.72 (0.63)
RD	0.055†	0.050^†	0.006	0.079**	0.219***	-0.233***	(0.69)	3.34 (0.76)
Des	0.359***	0.039	-0.050	0.255***	0.159***	0.002	0.024	3.42 (1.30)

^†^*p* < 0.10, *p < 0.05. **p < 0.01. ***p < 0.001. Prev, prevention regulatory focus; DT, desired tightness; CMD, civic moral disengagement; RD, reactions to normative daily transgressions; Des, social desirability; Edu, education level; Gen, gender. In bracket (Cronbach’s Alpha).

A subsequent one-way ANOVA was conducted in order to observe if there were differences in desired tightness across the Italian macro-regions (i.e., Northern, Central, Southern and Islands). This analysis revealed a significant but small difference: *F*_(2, 1178)_ = 5.07, *p* < 0.006. Southern Italy had the highest levels of desired tightness (*M* = 6.41, *SD* = 1.53), followed by Central Italy (*M* = 6.20, *SD* = 1.61) and Northern Italy (*M* = 5.86, *SD* = 1.40). Of course, results regarding Northern Italy should be interpreted with extreme caution given that it represented a very small percentage of the total sample.

**FIGURE 2:**
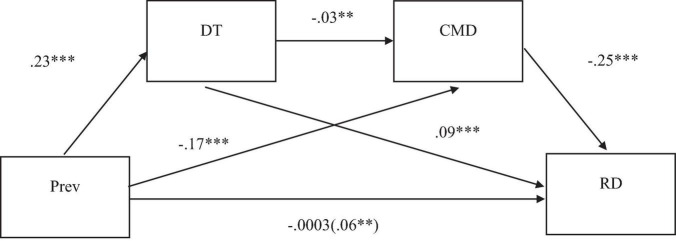
A serial mediation model showing the effects of prevention regulatory focus on reactions to daily normative transgressions sequentially via need for cognitive closure and desire for cultural tightness. All coefficients are unstandardized. **p < 0.01, ***p < 0.001. Total effect is displayed in parentheses. All effects were obtained controlling by social desirability. The covariate is not included. Prev, prevention regulatory focus; DT, desired tightness, CMD, civic moral disengagement; RD, reactions to normative daily transgressions.

The hypothesis was assessed via a serial mediation analysis (PROCESS Macro; Model 6; Hayes, 2022) with 5000 bootstrap samples and unstandardized regression coefficients. Social desirability was included in the model as covariate. Results are presented in Table 2 and Figure 2.

**TABLE 2:** Regression table.

				95% CI		
**Outcome**	**Predictor**	**Estimate**	**SE**	**Lower**	**Upper**	***p***
**Step 1**						
DT	Age	0.007	0.003	0.0005	0.0137	0.034
DT	Gen	0.305	0.097	0.114	0.497	0.001
DT	Edu	−0.075	0.05	−0.175	0.024	0.136
DT	Des	0.121	0.037	0.047	0.195	0.001
DT	Prev	0.238	0.059	0.121	0.354	<0.001
**Step 2**						
CMD	Age	−0.005	0.001	−0.008	−0.002	<0.001
CMD	Gen	−0.206	0.038	−0.281	−0.136	<0.001
CMD	Edu	−0.046	0.019	−0.085	−0.007	0.018
CMD	Des	0.056	0.014	0.027	0.085	<0.001
CMD	Prev	−0.174	0.023	−0.22	−0.129	<0.001
CMD	DT	−0.03	0.011	−0.052	−0.007	0.008
**Step 3**						
RD	Age	0.0007	0.001	−0.002	0.003	0.674
RD	Gen	−0.012	0.046	−0.104	0.08	0.798
RD	Edu	−0.004	0.024	−0.051	0.042	0.857
RD	Des	−0.006	0.018	−0.042	0.028	−708
RD	Prev	−0.0003	0.028	−0.057	0.056	0.992
RD	DT	0.094	0.013	0.067	0.121	<0.001
RD	CMD	−0.251	0.035	−0.321	−0.182	<0.001

Prev, prevention regulatory focus; DT, desired tightness; CMD, civic moral disengagement; RD, reactions to normative daily transgressions; Des, social desirability; Gen, gender; Edu, education.

As can be seen, controlling for social desirability, age, gender, and education level, desired tightness was positively and significantly predicted by prevention focus. Desired tightness was significantly and negatively associated with civic moral disengagement. Finally, civic moral disengagement was significantly and negatively associated with emotional reactions to normative transgression. The total effect of prevention focus on emotional reactions to normative transgression was significant. Moreover, the direct prevention focus effect became non-significant when the mediators were included in the model, thus indicating that the effect of prevention focus on emotional reactions to normative transgression was fully mediated by the mediators considered. Finally, and more importantly, the total and all specific prevention *indirect* effects through the mediators considered were significant (see Table 3).

**TABLE 3:** Indirect effects.

	Coefficient(β)	BootSE	Bootstrap 95% CI_s_
Prev → DT → RD	0.022	0.006	[0.045, 0.093]
Prev → CMD → RD	0.044	0.010	[0.025, 0.065]
Prev → DT → CMD → RD	0.001	0.0009	[0.0004, 0.003]
Total	0.068	0.029	[0.0105, 0.125]

Prev, prevention regulatory focus; DT, desired tightness, CMD, civic moral disengagement; RD, reactions to normative daily transgressions. Results controlling for social desirability, age, gender, and education level.

## Discussion

Why do people seemingly accept serious norm violations, as we have seen during the COVID pandemic? Examining this question was the scope of the present research. This question can be understood within an individual differences framework, given that people can act very differently—they can react positively, negatively, or indifferently—toward the same norm violations in the same social context. Furthermore, this research proposed a model in which a relatively general and chronic concern for negative outcomes, prevention regulatory focus, is exogenous to a relatively specific individual-level of desired cultural tightness.

Although this model has not yet been tested, the individual pathways are supported by previous research: for instance, Mula and Pierro (2022) found that the prevention focus and desired tightness are related; Di Santo et al. (2024) that desired tightness and moral disengagement are related; Fehr et al. (2019) that moral disengagement and reactions to norm violations are related. The hypothesized model was support and results were consistent with those uncovered by previous research. Specifically, a prevention focus was associated with high levels of desired tightness, desired tightness was associated with lower levels of civic moral disengagement, and civic moral disengagement was associated with lower levels of negative reactions to general norm violations. Consequently, there is evidence that these types of reactions can be indirectly explained by variation in a general individual difference characteristic, the prevention focus.

What can researchers do with this information? Although the model tested individual differences, it could be possible to at least temporarily raise (or lower) the prevention focus in individuals. In laboratory settings, this could be done via experimental primes. Although this could help establish the effect of experimentally manipulated prevention focus, it is unlikely to effect any kind of societal-level change. It could, however, be possible that publicity campaigns could highlight the general prevention focus, both in “micro-cultures” (e.g., a workplace or neighborhood) and in the general cultures (e.g., states and regions). In practice, enacting such campaigns is not a simple matter. Among other reasons, message recipients who perceive them as manipulative could react against them (e.g., psychological reactance, Miller et al., 2020). There is also some evidence that message recipients who are already high in prevention focus can, depending on the message design, can experience more psychological reactance (Kirmani and Zhu, 2007).

The hypothesized model was designed to be relatively general and, consequently, is not limited to any one topic. However, the model is relevant for both grand societal issues, for instance matters of procedural justice, as well as more focused behaviors, such as those effecting a workplace or neighborhood. A strength of this model is that it can be applied to various contexts and consequently add to these literatures.

There are also a number of limitations which should be discussed. Although there was a large sample, the study was entirely cross-sectional which strictly limits any claims of causality. This model could be replicated either in longitudinal designs or in a series of experimental designs that assess each individual pathway in the serial mediation model. Moreover, the measure of moral civic disengagement had elevated levels of skew and kurtosis. Consequently, future investigations could use other measures. An additional potential limitation is that our sample was collected in Italy. Readers may want to know if this model would replicate in other locations, such as the United States or non-WEIRD (Western, Educated, Industrialized, Rich, and Democratic) countries. Although this is ultimately an empirical question, being a general model, it is possible that individuals around that globe would respond similarly. However, this assumption, which is shared by many lines of research, needs to be put to the test in different populations. Finally, readers might be interested in the significant regional differences in desired tightness even though individuals’ desired tightness is theorized to be independent of the tightness of their locality. However, there are other factors which influence tightness, such as population density, which could not be taken into account in the present research. However, this question could be asked in a representative sample which was designed for these purposes.

## Conclusion

Based in the previous literature, this research tested the hypothesis that prevention focus would have an indirect effect on reactions to norm violations through desired cultural tightness and civic moral disengagement, such that prevention focus would be associated with higher levels of desired cultural tightness, desired cultural tightness would be associated with lower levels of civic moral disengagement, and civic moral disengagement would be associated with less negative reactions to norm violations. This hypothesis was supported in a large cross-sectional sample collected in Italy. Future research can assess this model in longitudinal and/or experimental designs, in different populations, and in reference to different behavioral domains.

## Data availability statement

The raw data supporting the conclusions of this article will be made available by the authors, without undue reservation.

## Ethics statement

The studies involving humans were approved by the Ethics Committee of the Department of Developmental and Social Psychology, Sapienza University of Rome. The studies were conducted in accordance with the local legislation and institutional requirements. The participants provided their written informed consent to participate in this study.

## Author contributions

CB: Writing – original draft. AP: Conceptualization, Data curation, Funding acquisition, Methodology, Project administration, Supervision, Writing – review and editing.

## References

[B1] Baldner C. Di Santo D. Viola M. Pierro A. (2022). Perceived COVID-19 threat and reactions to noncompliant health-protective behaviors: The mediating role of desired cultural tightness and the moderating role of age. *Int. J. Environ. Res. Public Health* 19:2364. 10.3390/ijerph19042364 35206549 PMC8871909

[B2] Bandura A. (2002). Selective moral disengagement in the exercise of moral agency. *J. Moral Educ.* 31 101–119. 10.1080/0305724022014322

[B3] Bandura A. Barbaranelli C. Caprara G. V. Pastorelli C. (1996). Multifaceted impact of self-efficacy beliefs on academic functioning. *Child Dev.* 67 1206–1222. 10.2307/1131888 8706518

[B4] Caprara G. V. Fida R. Vecchione M. Tramontano C. Barbaranelli C. (2009). Assessing civic moral disengagement: Dimensionality and construct validity. *Pers. Individ. Differ.* 47 504–509. 10.1016/j.paid.2009.04.027

[B5] Contu F. Di Santo D. Baldner C. Pierro A. (2023). Examining the interaction between perceived cultural tightness and prevention regulatory focus on life satisfaction in Italy. *Sustainability* 15:1865.

[B6] Di Santo D. Di Santo D. Pierro A. (2024). “Take Back the Land”: Analysis of the influence of environmental concern, cultural tightness, and moral disengagement on pro-environmental behavior intentions. *J. Commun. Appl. Soc. Psychol.* 34:e2760. 10.1002/casp.2760

[B7] Fehr R. Welsh D. Yam K. C. Baer M. Wei W. Vaulont M. (2019). The role of moral decoupling in the causes and consequences of unethical pro-organizational behavior. *Organ. Behav. Hum. Decis. Process.* 153 27–40.

[B8] Gelfand M. J. Jackson J. C. Pan X. Nau D. Pieper D. Denison E. (2021). The relationship between cultural tightness–looseness and COVID-19 cases and deaths: A global analysis. *Lancet Planet. Health* 5 e135–e144. 10.1016/S2542-5196(20)30301-6 33524310 PMC7946418

[B9] Gelfand M. J. Nishii L. H. Raver J. L. (2006). On the nature and importance of cultural tightness–looseness. *J. Appl. Psychol.* 91 1225–1244. 17100480 10.1037/0021-9010.91.6.1225

[B10] Gelfand M. J. Raver J. L. Nishii L. Leslie L. M. Lun J. Lim B. C. (2011). Differences between tight and loose cultures: A 33-Nation study. *Science* 332 1100–1104. 10.1126/science.1197754 21617077

[B11] Harrington J. R. Gelfand M. J. (2014). Tightness–looseness across the 50 united states. *Proc. Natl. Acad. Sci. U. S. A.* 111 7990–7995. 24843116 10.1073/pnas.1317937111 PMC4050535

[B12] Hartmann M. Müller P. (2023). Acceptance and adherence to COVID-19 preventive measures are shaped predominantly by conspiracy beliefs, mistrust in science and fear–A comparison of more than 20 psychological variables. *Psychol. Rep.* 126 1742–1783. 10.1177/00332941211073656 35212558 PMC8883133

[B13] Hayes A. F. (2022). *Introduction to Mediation, Moderation, and Conditional Process Analysis: A Regression-Based Approach*, 3rd Edn. New York, NY: Guilford Press.

[B14] Higgins E. T. Friedman R. S. Harlow R. E. Idson L. C. Ayduk O. N. Taylor A. (2001). Achievement orientations from subjective histories of success: Promotion pride versus prevention pride. *Eur. J. Soc. Psychol.* 31 3–23.

[B15] Higgins E. T. Shah J. Friedman R. (1997). Emotional responses to goal attainment: Strength of regulatory focus as moderator. *J. Pers. Soc. Psychol.* 72:515. 10.1037//0022-3514.72.3.515 9120782

[B16] Jackson J. C. Van Egmond M. Choi V. K. Ember C. R. Halberstadt J. Balanovic J. (2019). Ecological and cultural factors underlying the global distribution of prejudice. *PLoS One* 14:e0221953. 10.1371/journal.pone.0221953 31490981 PMC6730889

[B17] Kim H. Y. (2013). Statistical notes for clinical researchers: Assessing normal distribution (2) using skewness and kurtosis. *Restor. Dentistry Endodont.* 38 52–54. 10.5395/rde.2013.38.1.52 23495371 PMC3591587

[B18] Kirmani A. Zhu R. (2007). Vigilant against manipulation: The effect of regulatory focus on the use of persuasion knowledge. *J. Mark. Res.* 44 688–701.

[B19] Miller C. H. Massey Z. B. Ma H. (2020). “Psychological reactance and persuasive message design,” in *The Handbook of Applied Communication Research*, eds Dan O’Hair H. John O’Hair M. (Hoboken, NJ: Wiley), 457–483.

[B20] Mula S. Di Santo D. Gelfand M. J. Cabras C. Pierro A. (2021). The mediational role of desire for cultural tightness on concern with COVID-19 and perceived self-control. *Front. Psychol.* 12:713952. 10.3389/fpsyg.2021.713952 34594277 PMC8476743

[B21] Mula S. Pierro A. (2022). I don’t care why you do it, just don’t! Reactions to negative and positive organizational deviance partly depend on the desire for tightness of prevention-focused employees. *Front. Psychol.* 13:951852. 10.3389/fpsyg.2022.951852 36275286 PMC9583017

[B22] Pepitome A. (1981). The normative basis of agression: Anger and punitiveness. *Recherches Psychol. Soc. Paris* 3 3–17.

[B23] Pierro A. Kruglanski A. W. (2005). *Revised Need for Cognitive Closure Scale.* La Sapienza: Università di Roma.

[B24] Schoemann A. M. Boulton A. J. Short S. D. (2017). Determining power and sample size for simple and complex mediation models. *Soc. Psychol. Pers. Sci.* 8 379–386.

